# Aging of a Model Minority: A Diachronic Analysis of Two Quantitative Research Studies on Aging of Japanese in New York

**DOI:** 10.1093/geroni/igab046.890

**Published:** 2021-12-17

**Authors:** Itsuko Toyama, Taeko Nakashima

**Affiliations:** 1 St. Andrew's University, Souraku-gun, Kyoto, Japan; 2 Nihon Fukushi University, Handa-city, Aichi, Japan

## Abstract

This is a diachronic analysis of two quantitative research studies on the aging of Japanese and Japanese Americans living in Greater New York. How have older Japanese individuals, who once have been referred as “model minority,” lived and aged in Greater New York? All the data in this paper are based on the first research study conducted in 2006 and the second in 2018 (Ethical approval reference number 6, 2018). This paper reveals both the social transitoriness and the cultural immutability of the Japanese elderly community in Greater New York. The following is a summary of the findings: (1) a growing Japanese American community with US citizenship, higher academic qualification, and better communication competency has been observed. (2) The allowable range of private expense to hire personal caregivers has been widened. (3) Not only the concerns and anxieties for later lives but also the plans and preparations for aging are much the same. (4) The elderly are provided with culturally specific care (with regard to language, food, and concept of care)—even allowed to live with other Japanese people—and the needs of caregivers who can understand Japanese culture are satiated. (5) Almost half of those in the community find it difficult to eliminate the possibility of returning to Japan, and some of them have already chosen to migrate back to Japan.

